# A GC-MS Based Metabonomics Study of Rheumatoid Arthritis and the Interventional Effects of the Simiaowan in Rats

**DOI:** 10.3390/molecules201219776

**Published:** 2015-12-01

**Authors:** Yuming Wang, Xuejun Guo, Jiabin Xie, Zhiguo Hou, Yubo Li

**Affiliations:** 1 School of Traditional Chinese Materia Medica, Tianjin University of TCM, 312 Anshan West Road, Nankai District, Tianjin 300193, China; wangyuming226@163.com (Y.M.); guoxuejung666@126.com (X.G.); 18600893359@163.com (J.X.); hzgssh@163.com (Z.H.); 2 Tianjin State Key Laboratory of Modern Chinese Medicine, No. 88, Yuquan Road, Nankai District, Tianjin 300193, China

**Keywords:** GC-MS, metabonomics, rheumatoid arthritis, Simiaowan

## Abstract

Simiaowan (SMW) is a famous Chinese prescription widely used in clinical treatment of rheumatoid arthritis (RA). The aim of the present study is to determine novel biomarkers to increase the current understanding of RA mechanisms, as well as the underlying therapeutic mechanism of SMW, in RA-model rats. Plasma extracts from control, RA model, and SMW-treated rats were analyzed by gas chromatography coupled with mass spectrometry (GC-MS). An orthogonal partial least-square discriminant analysis (OPLS-DA) model was created to detect metabolites that were expressed in significantly different amounts between the RA model and the control rats and investigate the therapeutic effect of SMW. Metabonomics may prove to be a valuable tool for determining the efficacy of complex traditional prescriptions.

## 1. Introduction

Rheumatoid arthritis (RA) is a systemic inflammatory autoimmune disorder that is expressed as a symmetric polyarthritis associated with swelling and pain in multiple joints [1]. In ancient China, traditional Chinese medicine (TCM) has a long history of treatment for RA, which is recognized as a “bi” symptom [2,3]. Simiaowan (SMW) consists of four herbs, namely *Phellodendri Amurensis Cortex* (PAC), *Atractylodis Rhizoma* (AR), *Achyranthis Bidentatae Radix* (ABR), and *Coicis Semen* (CS), at a dose proportion of 2:1:1:2 [4]. SMW is widely recognized as a safe and effective prescription in the treatment of RA and other arthritis-related diseases [5,6]. However, the therapeutic efficacy of SMW against RA has not been investigated in a systematic study. Thus, identification of new biomarkers is still necessary to elucidate the underlying mechanisms of this disease.

Metabonomics is defined as the study of the endogenous metabolites present in an organism and the changes that occur in these compounds [7]. Metabonomics has been widely applied in many areas, including the diagnosis of disease, the discovery of mechanisms of drug action, and the toxicological studies [8,9,10,11]. Several analytical techniques, such as NMR, LC-MS, and GC-MS, its high sensitivity, good separation, and immediate identification of the separated metabolites have proven to be advantageous for the GC-MS method.

In the present study, a GC-MS based metabonomic approach was developed to screen and identify the metabolic perturbations associated with RA induced by *Freund*’*s* *complete* *adjuvant* (FCA) in RA-model rats. Orthogonal partial least-squares discriminant analysis (OPLS-DA) was carried out to discriminate the key differences between the RA model and the control rats. The aim of the present study is to characterize the biomarkers associated with RA and to investigate the therapeutic effects of SMW in RA-model rats using a metabonomic method.

## 2. Results and Discussion

### 2.1. Appearance and Histopathological Observation

Compared with the control rats, a few hours after FCA was injected into the right foot of the model rats, their right back feet began to swell continuously for (6 to 8) days, after this period, the swelling in the leg began to decrease, and the RA rats became lethargic and less active, in addition to losing body weight.

Typical pathological characteristics of synovial organization, such as synovial hyperplasia, swollen endothelial cells, and thickened endomembrane, were observed in RA rats, while endothelial dysfunction was not observed in the control rats. The symptoms were obviously alleviated when the RA rats were treated with SMW.

The above results show that the rat model of RA is successful, in accordance with the literature, and that SMW appears to have a therapeutic effect [12,13].

### 2.2. GC-MS Analysis of Plasma Samples

**Figure 1:**
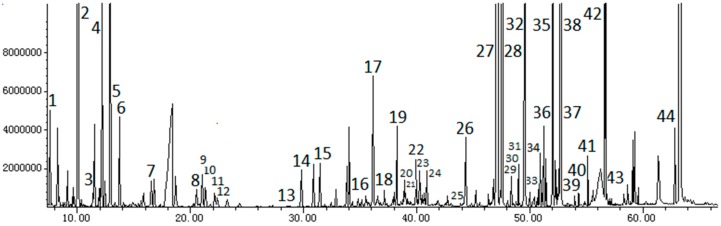
Total ion chromatogram (TIC) profile of metabolites in rat plasma by gas chromatography coupled with mass spectrometry (GC-MS).

Plasma samples from the control, model, and SMW groups were analyzed by GC-MS method as described above. Typical total ion chromatograms (TICs) are shown in Figure 1. Forty-four compounds were identified in the metabonomic profiling. The peaks considered to be representative chemical fingerprints of endogenous metabolites, which describe the metabolic perturbation induced by FCA and the protective effect of SMW on RA rats, are summarized in Table 1.

**Table 1:** The plasma metabolites of three groups of control, model and Simiaowan (SMW) identified by GC-MS. (**A**: control group; **B**: model group; **C**: SMW group).

Code	RT (min)	Compounds	VIP	Mean ± SD		
				A	B	C
1	7.566	Acetamide	0.90	3.83 ± 0.18	3.83 ± 0.15	3.94 ± 0.13
2	10.038	Propionic acid	0.84	6.82 ± 1.56	7.64 ± 2.07	4.91 ± 0.45
3	11.508	l-alanine	1.77	4.27 ± 0.52	5.29 ± 0.81 *	3.70 ± 0.92 **^#^**
4	12.081	l-glycine	1.70	2.85 ± 0.21	3.48 ± 0.60 *	1.97 ± 0.40 **^#^**
5	12.901	Oxalic acid	0.98	4.28 ± 0.83	3.88 ± 0.71	4.35 ± 1.36
6	13.724	Butyric acid	1.17	0.63 ± 0.37	0.46 ± 0.18 *	1.02 ± 0.31 **^#^**
7	16.520	l-valine	0.88	2.06 ± 0.37	2.27 ± 0.44	1.72 ± 0.34
8	20.549	l-Leucine	1.10	2.32 ± 0.50	2.61 ± 0.47 *	2.11 ± 0.39 **^#^**
9	21.048	Phosphoric Acid	0.97	3.21 ± 0.32	3.11 ± 0.44	2.62 ± 0.32
10	21.325	glycerine	0.94	0.80 ± 0.14	0.77 ± 0.24	0.86 ± 0.43
11	22.158	l-proline	0.98	8.58 ± 1.01	8.91 ± 0.70	7.98 ± 0.72
12	22.391	l-isoleucine	1.20	1.29 ± 0.26	1.49 ± 0.23 *	1.06 ± 0.22 **^#^**
13	28.615	2,3-dihydroxy butyric acid	0.85	0.06 ± 0.01	0.06 ± 0.01	0.05 ± 0.01
14	29.813	l-serine	0.95	1.93 ± 0.36	2.05 ± 0.25	1.46 ± 0.37
15	31.448	l-threonine	1.37	0.96 ± 0.12	1.09 ± 0.13 *	0.75 ± 0.12 **^#^**
16	35.589	Malic acid	0.86	0.08 ± 0.02	0.09 ± 0.04	0.05 ± 0.02
17	36.482	l-aspartate	1.46	0.15 ± 0.04	0.19 ± 0.05 *	0.09 ± 0.02 **^#^**
18	37.153	Creatinine	0.92	0.81 ± 0.44	0.76 ± 0.49	0.49 ± 0.42
19	38.994	l-Phenyl Alanine	1.41	0.75 ± 0.12	0.85 ± 0.10 *	0.58 ± 0.11 **^#^**
20	39.007	Glutaminate	0.84	0.52 ± 0.12	0.49 ± 0.10	0.40 ± 0.08
21	39.120	Arbinofuranose	0.89	0.06 ± 0.02	0.06 ± 0.03	0.05 ± 0.01
22	40.142	l-Asparagine	0.92	0.07 ± 0.02	0.07 ± 0.01	0.05 ± 0.01
23	40.678	d-fructose	1.41	0.34 ± 0.17	0.25 ± 0.07 *	0.25 ± 0.06
24	40.899	l-lysine	0.95	0.72 ± 0.20	0.89 ± 0.52	0.53 ± 0.16
25	43.900	l-ornithine	0.82	0.09 ± 0.09	0.09 ± 0.07	0.08 ± 0.03
26	44.329	Tetradecoic acid	0.84	1.12 ± 0.20	1.09 ± 0.14	1.04 ± 0.20
27	45.565	d-mannose	2.16	0.71 ± 0.07	0.93 ± 0.14 *	0.93 ± 0.32
28	47.545	d-glucose	0.90	55.04 ± 7.82	54.08 ± 9.30	50.88 ± 4.87
29	48.365	d-galactose	0.93	14.7 ± 3.42	13.55 ± 3.36	11.59 ± 1.86
30	48.995	Glucitol	0.87	0.56 ± 0.11	0.53 ± 0.11	0.43 ± 0.09
31	49.550	Hexadecanoic Acid	0.92	14.47 ± 4.60	13.75 ± 3.20	13.65 ± 3.81
32	49.991	β-d-glucopyranose	0.83	0.31 ± 0.11	0.32 ± 0.12	0.28 ± 0.06
33	50.925	Inositol	0.85	0.59 ± 0.11	0.66 ± 0.17	0.40 ± 0.05
34	51.240	heptadecanoic acid	0.91	0.81 ± 0.21	0.77 ± 0.11	0.75 ± 0.18
35	52.262	Linoleic acid	1.69	0.36 ± 0.13	0.21 ± 0.10 *	0.51 ± 0.25 **^#^**
36	52.433	Oleinic acid	1.45	0.47 ± 0.19	0.30 ± 0.16 *	0.57 ± 0.25 **^#^**
37	52.607	l-tryptophane	1.26	2.01 ± 0.55	1.91 ± 0.43 *	1.64 ± 0.46
38	52.741	Stearic acid	0.89	27.01 ± 4.75	27.42 ± 3.49	26.54 ± 4.60
39	53.964	Nonadecanoic acid	0.87	0.07 ± 0.01	0.07 ± 0.01	0.06 ± 0.01
40	54.311	Arachidonic acid	1.75	0.10 ± 0.02	0.08 ± 0.01 *	0.09 ± 0.03
41	55.124	Eicosanoic acid	0.93	0.33 ± 0.08	0.32 ± 0.05	0.30 ± 0.07
42	56.814	2,3-Dihydroxy Hexadecanoic acid	0.92	0.18 ± 0.04	0.20 ± 0.02	0.19 ± 0.03
43	57.193	Docosanoic acid	0.85	0.03 ± 0.01	0.03 ± 0.01	0.03 ± 0.01
44	62.851	Cholesterol	1.18	0.71 ± 0.14	0.65 ± 0.14 *	0.68 ± 0.17

Data are expressed as mean ± S.D.; *: Significantly different from control; **^#^**: Significantly different from model.

### 2.3. Identification of Potential Biomarkers

A list of 15 variables representing individual metabolites that were tagged as potential biomarker candidates was generated according to the results of the OPLS-DA and SPSS analyses. Eight of these metabolites, including l-alanine, l-leucine, l-isoleucine, l-glycine, l-threonine, l-aspartate, l-phenylalanine, and d-mannose, were significantly increased in the RA-model rats. In contrast, seven metabolites—butyric acid, d-fructose, linoleic acid, oleinic acid, l-tryptophan, arachidonic acid, and cholesterol—were found at lower levels. The related pathway of each biomarker was recorded by searching the KEGG pathway database (http://www.genome.jp/kegg/). The identified metabolites and their possible related pathways are summarized in Table 2.

**Figure 2:**
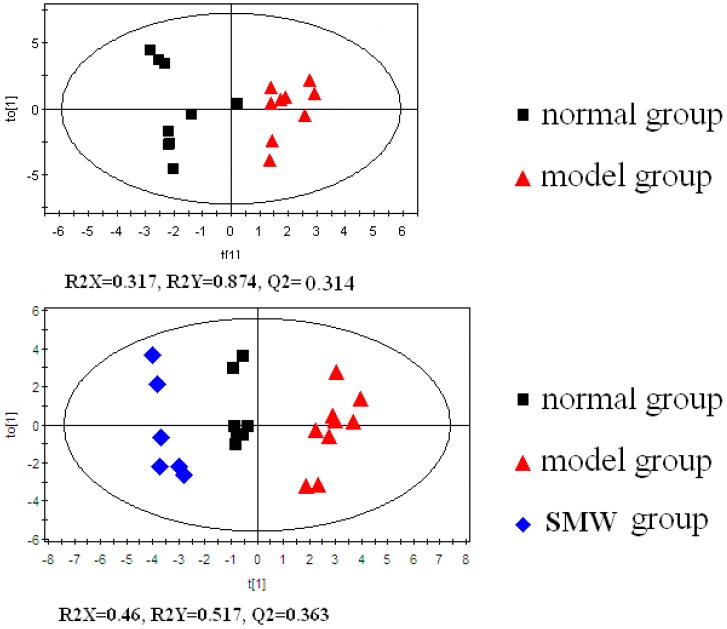
The score plot from the orthogonal partial least-square discriminant analysis (OPLS-DA) of the GC-MS data obtained from the plasma samples of normal rat, rheumatoid arthritis (RA) rats, and SMW-treated rat.

**Figure 3:**
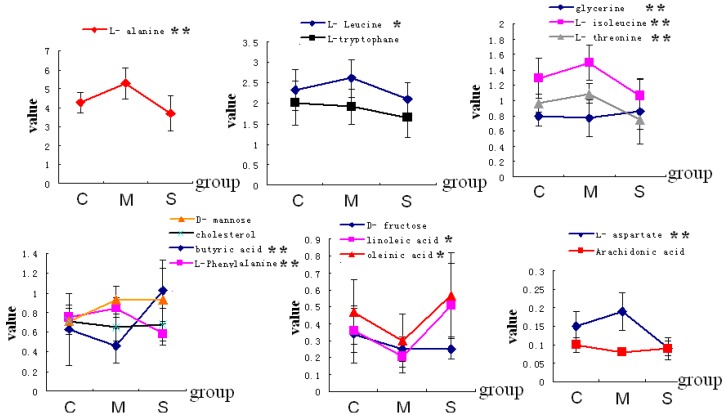
The change trend graph of metabolites (*: *p* < 0.05; **: *p* < 0.01).

OPLS-DA is a supervised statistical method that finds directions in a multivariate space to ensure the maximum separation of observations belonging to different classes. After unit variance (UV) scaling, OPLS-DA was used to obtain a better understanding of the different plasma-metabolite patterns in different groups. Figure 2 shows the score plot of the OPLS-DA model, which clearly discriminated the normal group from both the model and the SMW groups. R2 estimates goodness of fit while Q2 is an estimate of the predictive ability of the model. More important, Q2 is an index of the similarity of the observations in each group. The results revealed that the model is a very excellent model. The results showed that each groups were gathered together. The SMW group cluster together on the left hand side of the data set and the model group on the right hand side of the data set. They were all separated from the normal group which was on the middle of the data set. We can see from this data that the GC/MS system was capable of clearly differentiating the bile from different groups. In addition, amino acid l-glycine and fatty acids, such as butyric, linoleic, and oleinic acids, showed a subtle decrease in the RA group and reverted to control levels in the SMW group (Figure 3).

**Table 2:** Metabolites of variable importance for projection (VIP) > 1 in normal group and model group.

Retention Time	Metabolites	RA Group Compared with Control Group	Metabolic Pathway
11.508	l-alanine	↑	Alanine, aspartate and glutamate metabolism
12.081	l-glycine	↑	Glycine, serine and threonine metabolism
13.724	Butyric acid	↓	
20.549	l-leucine	↑	Valine, leucine and isoleucine metabolism
22.391	l-isoleucine	↑	Valine, leucine and isoleucine metabolism
31.448	l-threonine	↑	Glycine, serine and threonine metabolism
36.482	l-aspartate	↑	Alanine, aspartate and glutamate metabolism
38.944	l-phenylalanine	↑	Phenylalanine metabolism
40.678	d-fructose	↓	Fructose and mannose metabolism
45.565	d-mannose	↑	Fructose and mannose metabolism
52.262	Linoleic acid	↓	Lipid metabolism
52.433	Oleinic acid	↓	Lipid metabolism
52.607	l-tryptophan	↓	Tryptophan metabolism
54.311	Arachidonic Acid	↓	Arachidonic acid metabolism Lipid metabolism
62.851	Cholesterol	↓	Lipid metabolism

### 2.4. Biological Interpretation

In this study, possibly important potential biomarkers in the RA and SMW groups were found using a metabonomic approach (Table 2). The results effectively indicate that the metabolites identified are biomarkers of the anti-arthritic effect related to the action mechanism of SMW.

#### 2.4.1. Amino Acid Metabolism

In the present study, the concentrations of some branched-chain amino acids (BCAA), such as l-alanine, l-leucine, and l-isoleucine, increased in the RA rats, whereas they decreased in the SMW group. BCAAs have been shown to increase the production of cytokines, including interleukins 1 and 2, tumor necrosis factor, and interferon [14,15]. Cytokines play a fundamental role in the inflammation, articular destruction, and comorbidity associated with RA [16,17]. The effects of SMW on amino acid metabolism in RA rats suggest that SMW inhibits protein breakdown and degradation of joint collagen.

The results show that l-threonine, l-aspartic acid, and l-phenylalanine are significantly decreased in the SMW group compared with the model group. The above results show that SMW can mainly enhance immunity, inhibit proliferation of cell factors, reduce active oxygen metabolism, and so on.

Kynurenine (Kyn) is closely related to the various kinds of chronic diseases. Up-regulation of Kyn in RA rats may be induced by excessive activation of tryptophan 2,3-dioxygenase (TPHD) in the metabolic pathway producing *N*′-formylkynurenine and activating Kyn formamidase, which generates Kyn in the RA process and directly causes abnormalities Trp metabolism. SMW was observed to prevent the increased tendency of Kyn and decreased tendency of Trp in the RA process, indicating that the therapeutic effects of SMW may be based on the regulation of the TPHD dysfunction in Trp metabolism.

#### 2.4.2. Lipid Metabolism

The decrease in glycerol concentrations in RA rats coincides with the decreased body-fat deposition and/or increased fat consumption. Inflammation has been reported to impair reverse cholesterol transport (RCT), a major atheroprotective function of HDL, where cholesterol movement from macrophage to plasma is reduced, leading to a decreased cholesterol level in the plasma of the inflammation model [18]. As an important component of the lipid, RA activity inflammation may reflect the reduced capacity of inflammatory HDL particles to efficiently accept cholesterol from macrophages. The administration of SMW leads to an increase in the cholesterol level, indicating the recovered synthesis and transport of cholesterol, which may be related to the recovery of RCT as described above.

#### 2.4.3. Energy Metabolism

Decreased levels of linoleic and oleinic acids were observed in RA rats compared with the control. Linoleic acid is a kind of essential fatty acid in the body that can generate arachidic acid (AA) by the Δ ^6^-dehydrogenase and Δ ^5^-dehydrogenase function. AA, a polyunsaturated essential fatty acid, is well known for mediating inflammation. After reacting with an enzyme called cyclooxygenase or lipoxygenase, AA generates eicosanoids, such as prostacyclin, TXA2, leucotriene, and so on, which is a kind of immune response to inflammation, and plays the role of signal transmission molecule. This response may be due to the hypoxic environment in RA joints, which is consistent with the NMR evidence of lipolysis in SF compared to that in serum [19]. Overall, the alteration in the fatty acid profile in the RA group may partly explain the increased synthesis of eicosanoids.

After the administration of SMW, the fatty acid level was significantly increased, while pro-inflammatory eicosanoid levels were reduced, resulting in a reduction in inflammation. Figure 3 shows that, after the administration of SMW, the values of these metabolite levels had tended to return from the model group to the control group. SMW restores the fatty acid metabolism network to its normal state by regulating some key enzymes, including PLA2, which supports our results on AA metabolism.

### 2.5. Mechanism Supposition of SMW

SMW intervention of RA treated rats tended to bring levels of l-alanine, l-leucine, l-isoleucine, l-glycine, l-threonine, l-aspartate, d-mannose, l-phenylalanine, butyric acid, d-fructose, linoleic acid, oleinic acid, l-tryptophan, arachidonic acid, and cholesterol back to normal.

In the present study, an increased level of AA and a decreased level of linoleic acid were observed in RA group, which can be attributed to the large amount of linoleic acid consumed during the formation of AA. The results indicate that SMW may suppress Δ ^6^-dehydrogenase, Δ ^5^-dehydrogenase, and COX-2 expression, restrain arachidonic acid metabolism, and reduce inflammation.

The activity of glyceraldehyde-3-phosphate dehydrogenase and lactate dehydrogenase in RA synovial cells has been reported to be higher than that incompared with normal synovial cells, indicating that glycolysis is increased in the RA synovial membrane, which reduces sugar concentrations [20]. The effect of SMW on sugar metabolism, as observed in the present study, suggests an improvement in the symptoms of RA; this result is consistent with those in the literature [8].

The subjects of our study were male Wistar rats, and the metabolic manifestation of human disease was probably not fully simulated by the animal models created. Therefore, further research is necessary to identify the biomarkers and anti-RA mechanism of SMW for clinical applications.

## 3. Experimental Section

### 3.1. Materials and Reagents

PAC was purchased from the Sichuan Hehuachi herbal market (Sichuan, China). AR, ABR, and CS were purchased from a Tianjin herbal factory (Tianjin, China). All herbs were identified at the Tianjin University of TCM and sun-dried thoroughly.

*N*,*O*-bis(trimethylsilyl)trifluoroacetamide (BSTFA), trimethylchlorosilane (TMCS), decanoic acid, methylhydroxylamine hydrochloride, and pyridine were of GC grade and purchased from Fluka (Buchs, Switzerland). FCA was provided by Sigma Co. (St. Louis, MI, USA). All other reagents were of analytical grade.

### 3.2. Preparation of SMW

SMW was prepared using the recipe provided in the Chinese Pharmacopoeia [4]. PAC (250 g), AR (125 g), ABR (125 g), and CS (250 g) were chopped into small pieces and ground well. The powdered herbs were combined and dissolved in distilled water at a concentration of 1 g raw material/mL and stored in a refrigerator at 4 °C until administration to animals.

### 3.3. RA Model and Drug Administration

Eighteen male Wistar rats of approximately (200 ± 20) g were purchased from the Animal Center of the Tianjin University of TCM (Tianjin, China). After an initial acclimation period of 1 week in cages, all rats were randomly divided into three separate groups: the control group, the model group, and the SMW group (4.32 g/kg/day). All rats, except those in the control group, were injected with 0.1 mL FCA in the rear right foot to induce inflammation. On the 9th day after immunization, appropriate medicines were provided to the SMW group by intragastric administration once daily for 14 days. The rats in both the model and the control groups were fed with an equal volume of distilled water. The animals were killed 2 h post-administration on the 22nd day, and plasma was quickly collected and stored at −80 °C [21,22].

### 3.4. Preprocessing of Samples

A 100 μL plasma was spiked with an internal standard (IS) solution (20 μL of 0.10 mg/mL decanoic acid); 300 μL of ACN was then added to precipitate the proteins. After centrifugation at 10,000× *g* for 10 min, 200 μL volume of the supernatant was transferred to a 2 mL GC vial and evaporated to dryness under a stream of nitrogen gas. Methylhydroxylamine hydrochloride (50 μL, 15 mg/mL in pyridine) was added to the vial, and the methoxamination reaction was allowed to proceed at 70 °C for 1 h. A 50 μL volume of BSTFA (containing 1% TMCS) was subsequently added, and the reaction was allowed to proceed at 70 °C for 1 h. A 300 μL volume of *n*-heptane (containing 0.10 mg/mL of docosane, reference compound) was added next, and the sample was vortexed for 1 min to ensure complete mixing prior to analysis [23,24,25].

### 3.5. GC–MS Analysis

The derivatized extracts were analyzed on Agilent 6890N gas chromatography coupled with 5973 mass spectrometric detector. A 1 μL aliquot was injected into a HP-5MS capillary column (30 m × 250 μm i.d., 0.25 μm) in the pulsed splitless mode. The injection temperature and the interface temperature were set to 280 °C. The initial GC oven temperature was kept at 70 °C for 5 min, then increased to 80 °C at 10 °C/min, maintained for 2 min, and then increased to 100 °C at 5 °C/min and maintained for 15 min, increased by 6 °C/min to 180 °C and held for 6 min, and then finally increased to 290 °C at 8 °C/min, which was held for 5 min. The carrier gas was helium at a flow of 1.0 mL/min. The solvent delay was set for 8 min. The measurements were made with electron impact (EI) ionization (70 eV) in the full scan mode (45 *m*/*z* to 600 *m*/*z*). The NIST mass spectra and Wiley libraries were searched to identify the compounds.

### 3.6. Data Processing and Pattern Recognition

Peaks with intensities higher than threefold of the signal-to-noise (S/N) ratio and consistently present in each sample were recorded. All known artifact peaks, such as peaks due to column bleed and BSTFA artifact peaks, were excluded from the following data analysis.

Before multivariate analysis, each peak area was normalized using IS to ensure that each sample was represented by a collection of the variables to characterize its biochemical pattern. All the compounds were analyzed by matching against compounds available in the NIST and the Wiley libraries. OPLS-DA was used for the analysis of metabolite profiles using the SIMCA-P, version 12.0, software (Umetrics AB, Umea, Sweden). The metabolites were selected as candidates when their variable importance for projection (VIP) values were larger than 1.0. The significance of the between-group differences for these metabolites was examined by the student’s *t*-test using SPSS 12.0 (SPSS Inc., Chicago, IL, USA). P-values less than 0.05 were taken to indicate statistical significance.

## 4. Conclusions

In this study, a novel method based on GC-MS and OPLS-DA was developed for the metabolic profiling of FCA-induced RA in rats and determination of the effect of SMW intervention. Candidate biomarkers of the anti-RA effect of SMW were identified. The present work shows that the metabonomic approach is a potentially powerful tool for evaluating the pharmacological effects and mechanisms of complex traditional prescriptions.
